# Mitral valve surgery post-transcatheter aortic valve replacement: A 10-year, single-center, retrospective analysis

**DOI:** 10.1016/j.xjse.2025.100063

**Published:** 2025-07-16

**Authors:** Marco Tagliafierro, Rahul Kanade, Isao A. Anzai, Fady K. Soliman, Farooq Mirza, Arnar Geirsson, Paul Kurlansky, Michael Argenziano, Isaac George, Luigi Pirelli

**Affiliations:** Division of Cardiothoracic Surgery, Department of Surgery, Columbia University Medical Center, Columbia University College of Physicians and Surgeons, NewYork-Presbyterian Hospital, New York, NY

**Keywords:** mitral valve, TAVI, surgery post-TAVR, transcatheter aortic valve replacement, post-TAVR mitral valve surgery

## Abstract

**Objective:**

Cardiac operations after transcatheter aortic valve replacement (TAVR) are complex procedures with high morbidity and mortality. We sought to determine the outcomes of mitral valve surgery in patients with previous TAVR.

**Methods:**

Among 2339 patients who underwent mitral valve surgery at our institution between July 2014 and July 2024, a retrospective, descriptive analysis was performed on 19 consecutive patients with a history of TAVR requiring mitral valve surgery.

**Results:**

Mean age was 73.1 ± 9.5 years. The median (interquartile range) time since TAVR was 1 year (1, 3). The indication for mitral valve surgery was active infective endocarditis in 4 patients (21%), and degenerative disease in the remaining. The average Society of Thoracic Surgeons predicted risk of mortality was 12.0 ± 9.3%, with 6 (31.6%) urgent and 2 (10.5%) emergent operations. Nine patients (47.3%) had history of previous cardiac surgery. Mitral valve repair was performed in 1 patient, whereas replacement with a bioprosthetic valve in all other cases. Transcatheter valve explant was necessary in 10 operations (52.6%), and 13 patients had a concomitant procedure. The primary end point of all-cause mortality at 30 days was met in 5 patients (26.3%), 3 of whom underwent TAVR explant. One patient (5.3%) suffered a stroke. Median crossclamp and cardiopulmonary bypass times were 144 (106, 189) and 214 (155, 253) minutes, respectively. Postoperative dialysis was required in 4 (21.1%). One patient required a reintervention and 3 were re-hospitalized after discharge.

**Conclusions:**

Mitral valve surgery in the presence of TAVR is uncommon but carries a high risk of mortality and morbidity.

## Graphical abstract

**Figure:**
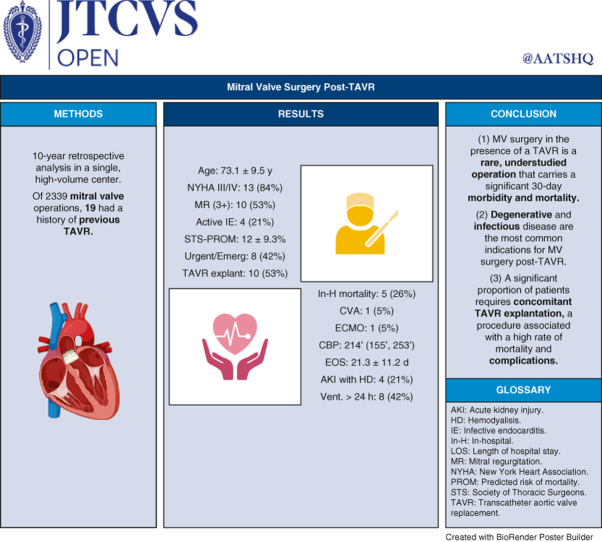

**Figure:**
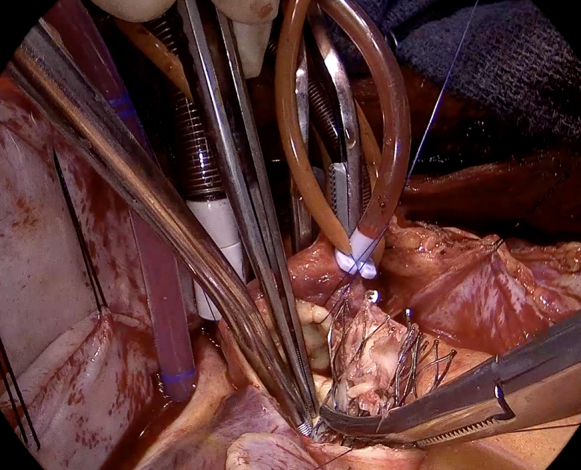
TAVR explant is associated with significant morbidity and mortality.

**Central Message**

Mitral valve surgery in patients with previous TAVR is a challenging, understudied operation that carries significant postoperative morbidity and mortality.

**Perspective**

Rates of surgical mitral valve interventions in the presence of a transcatheter aortic valve replacement (TAVR) will likely increase in the near future, as TAVR is becoming the preferred treatment strategy in patients with aortic valve stenosis. It portends high mortality and morbidity, especially if combined with TAVR explantation.

Transcatheter aortic valve replacement (TAVR) has become the preferred strategy for treatment of severe aortic valve (AV) stenosis. Patients with progressive mitral valve (MV) disease that was not significant at the time of the TAVR, MV disease that did not regress after AV treatment, or patients with de novo pathology such as infective endocarditis (IE) might require further MV interventions.^1^

An abnormal MV that shows moderate degree of disease is often left untreated at the time of the TAVR.^2^ The MV can exhibit abnormal degenerative or functional features, and the disease can progress over time as a result of either primary valve pathology or worsening heart function, leading to a more significant degree of disease.^3^ This can be related to chordal rupture in myxomatous prolapsing valves, untreated coronary disease and left ventricle dilation in ischemic secondary mitral regurgitation (MR), or longstanding atrial fibrillation leading to functional atrial insufficiency.^4^ Moreover, the MV may show stigmata of mild rheumatic disease at the time of TAVR that progresses over time to become severely dysfunctional with high transvalvular gradients and/or associated regurgitation. The American College of Cardiology/Transcatheter Valve Therapy and the European Society of Cardiology/European Association for Cardiothoracic Surgery have provided recommendations for MV therapy independent of the presence of a prosthetic AV, be it surgical or transcatheter.^5^^,^^6^

Current transcatheter therapies for treatment of MV pathologies, including mitral transcatheter edge-to-edge repair and MV replacements (transcatheter mitral valve replacement [TMVR]), are not always available as the result of poor anatomy, MV calcific disease, high predicted risk or existing mitral stenosis (MS), risk of left ventricular outflow tract obstruction (LVOTO), and inadequate size of available devices.^7^, ^8^, ^9^ In addition, there is no commercially available TMVR in the United States at the current time. There remains an important clinical need for open surgery in patients who need mitral interventions in which no transcatheter option is available.

De novo pathologies of the MV are equally common in patients with previous TAVR. IE of the native MV is frequently considered for surgical therapy.^10^ Moreover, an infection primarily affecting the aortic transcatheter heart valve (THV) can spread to the MV through the aorto-mitral continuity.^11^

Open-heart cardiac surgery in patients with a history of TAVR is associated with greater mortality and morbidity.^12^^,^^13^ Technical challenges include the exposure of the MV, deformation of the TAVR stent frame during left atrial retraction, and the potential need for explantation of THV devices. The outcomes of surgery are the results of longer and technically more difficult operations that can involve isolated MV intervention or combined aortic and mitral surgery. As of yet, very limited information is known about technical considerations and outcomes of MV surgery post-TAVR. We thereby report our single-center experience of patients with history of TAVR who underwent MV surgery at New York Presbyterian Hospital/Columbia University Medical Center.

## Methods

### Patient Population and Data Collection

**Figure 1:**
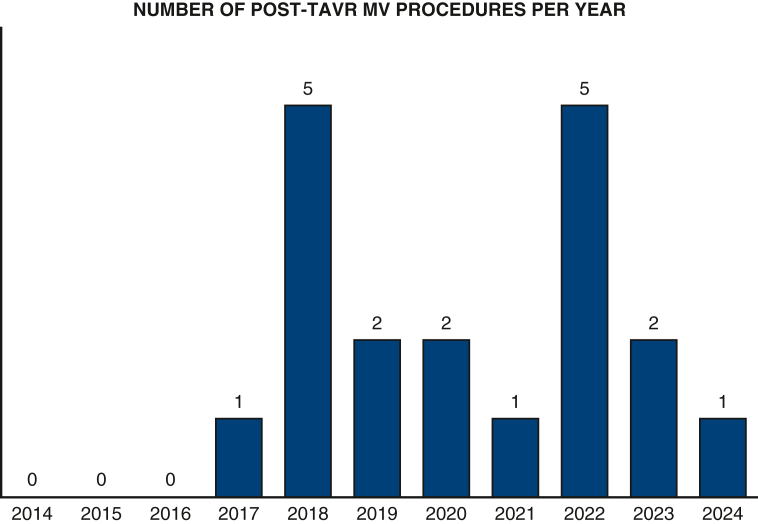
Number of mitral valve (MV) surgeries per year from July 1, 2014 to July 1, 2024. *TAVR*, Transcatheter aortic valve replacement.

After institutional review board approval (number: AAAV5722, approved on November 7, 2024), we retrospectively collected data from patients with a history of previous TAVR who underwent MV surgery at Columbia University Medical Center, New York, between July 1, 2014, and July 1, 2024. This comprised 19 MV operations in patients with no previous MV intervention at the time of the TAVR (Figure 1). Indication for MV therapy at the current time was determined via clinical and echocardiographic assessment.^14^ Clinical data were collected from chart review; therefore, individual consent was not required.

### Primary and Secondary End Points

The primary end points were defined as all-cause mortality and stroke at 30 days. Secondary end points included crossclamp and cardiopulmonary bypass times (XCT and CPB, respectively), intensive care unit (ICU) and in-hospital lengths of stay, postoperative renal failure requiring dialysis, prolonged ventilation (>24 hours), rate of reintervention attributable to cardiac causes, and rate of rehospitalization.

### Statistical Analysis

Continuous variables were checked for normality of distribution using the Shapiro-Wilk test: they are expressed as mean ± standard deviation when normally distributed, otherwise as median (interquartile range), as appropriate. Categorical variables are reported as absolute numbers and percentages. Calculations of predicted risk of outcomes for each surgery were performed using the “STS Adult Cardiac Surgery Database Operative Risk Calculator” (app version 2.0.5),^15^ or, whenever a concomitant AV operation was performed, on the “STS AVR + MVRr ± CABG Operative Risk Calculator” (app version 1.0.0).^16^ Of note, in the app version 2.0.5 of the “STS Adult Cardiac Surgery Database Operative Risk Calculator,” we were not able to specify TAVR as a previous intervention.

## Results

### Baseline Patient Characteristics

A total of 19 patients were included in the analysis (Table 1). Mean age was 73.1 ± 9.5 years, and 7 were male. All patients had severe MV pathology with indication for surgical intervention. The majority had signs of primary degenerative MV disease (94.7%), and only 1 patient had functional MR. Seven patients (36.8%) had evidence of IE. Twelve patients presented with significant mitral stenosis, with mean mitral transvalvular gradients of 6.9 ± 2.5 mm Hg; more precisely, 3 patients had severe MS (mean transvalvular gradient >10 mm Hg), and 9 had moderate MS (mean transvalvular gradient between 5-10 mm Hg). MR was moderate in 2 patients (10.5%) and severe in 8 (42.1%). All 19 patients had a previous TAVR, 15 (78.9%) with a balloon-expandable and 4 (21.1%) with a self-expandable valve. The THV was implanted a median of 1 (1, 3) year before the MV surgery. Preoperative transesophageal echocardiography and computed tomography scan showed moderate or greater aortic stenosis (AS) in 2 patients (10.5%), whereas 6 (31.6%) had mild and 2 (10.5%) had moderate intravalvular THV aortic regurgitation (AR): of these, 4 had an active infectious process affecting also the TVH. There were 3 patients who had severe TR and 2 (10.5%) had evidence of significant coronary artery disease. Mean preoperative Society of Thoracic Surgeons predicted risk of mortality was 12.0 ± 9.3%. One patient had end-stage renal disease requiring renal-replacement therapy.

**Table 1:** Descriptive analysis of preoperative variables

Variable	Value
Age, y, mean ± SD	73.1 ± 9.5
Male gender, n (%)	7 (36.8)
Cardiovascular risk factors, n (%)	
Hypertension	16 (84.2)
Tobacco use (former or current)	7 (36.8)
Diabetes mellitus	6 (31.6)
CAD	7 (36.8)
Sleep apnea	2 (10.5)
BMI, kg/m^2^, mean ± SD	28.8 ± 6.9
COPD, n (%)	8 (42.1)
Home oxygen required	3 (15.8)
Renal function	
Preoperative creatinine, mg/dL, median (IQR)	0.9 (0.7, 1.2)
Preoperative dialysis, n (%)	1 (5.3)
Cerebrovascular disease, n (%)	8 (42.1)
Endocarditis, n (%)	
Treated	2 (10.5)
Active	4 (21.1)
Liver disease, n (%)	2 (10.5)
Previous myocardial infarction, n (%)	1 (5.3)
Hx of recent mediastinal radiation, n (%)	2 (10.5)
LVEF, median (IQR)	60 (55, 64)
Preserved (>50%), n (%)	16 (84.2)
NYHA class, n (%)	
I	1 (5.3)
II	2 (10.5)
III	8 (42.1)
IV	5 (26.3)
Primary mitral valve disease, n (%)	26 (94.7)
Secondary mitral valve disease, n (%)	1 (5.3)
Mitral stenosis, n (%)	12 (63.2)
Mean transvalvular gradient, mm Hg, mean ± SD	6.9 ± 2.5
Trivial/trace	0 (0)
Mild	3 (15.8)
Moderate	4 (21.1)
Severe	5 (26.3)
Mitral regurgitation, n (%)	
Trivial/trace	2 (10.5)
Mild	7 (36.8)
Moderate	2 (10.5)
Severe	8 (42.1)
Aortic stenosis, n (%)	2 (7.4)
Mean transvalvular gradient, mm Hg, mean ± SD	26 ± 2
Trivial/trace	0 (0)
Mild	0 (0)
Moderate	1 (5.3)
Severe	1 (5.3)
Aortic regurgitation, n (%)	
Trivial/trace	4 (21.1)
Mild	6 (31.6)
Moderate	2 (10.5)
Severe	0 (0)
Tricuspid regurgitation, n (%)	
Trivial/trace	6 (31.6)
Mild	2 (10.5)
Moderate	6 (31.6)
Severe	3 (15.8)
Previous cardiac surgeries, n (%)	
TAVR	19 (100)
Balloon-expandable TAVR	16 (84.2)
Self-expandable TAVR	5 (26.3)
Years of TAVR implant before MV surgery, median (IQR)	1 (1, 3)
PCI	5 (26.3)
CABG	1 (5.3)
ViV post-SAVR	1 (5.3)
Other	5 (26.3)
STS operative mortality predicted risk, %, mean ± SD	12.0 ± 9.3
STS morbidity and mortality predicted risk, %, mean ± SD	29.6 ± 16.4
STS stroke predicted risk, %, mean ± SD	3.2 ± 2.2
STS renal failure predicted risk, %, mean ± SD	10.1 ± 9.9
STS re-operation predicted risk, %, mean ± SD	6.9 ± 3.9
STS prolonged ventilation predicted risk, %, mean ± SD	19.9 ± 13.0
STS deep sternal wound infection predicted risk, %, median (IQR)	0.055 (0.051, 0.084)
STS long hospital stay (>14 d) predicted risk, %, mean ± SD	25.5 ± 14.5
STS short hospital stay (<6 d) predicted risk, %, mean ± SD	11.3 ± 7.4

*SD*, Standard deviation; *CAD*, coronary artery disease; *BMI*, body mass index; *COPD*, chronic obstructive pulmonary disease; *IQR*, interquartile range; *Hx*, history; *LVEF*, left ventricular ejection fraction; *NYHA*, New York Heart Association; *TAVR*, transcatheter aortic valve replacement; *MV*, mitral valve; *PCI*, percutaneous coronary intervention; *CABG*, coronary artery bypass graft; *ViV*, valve-in-valve; SAVR, surgical aortic valve replacement; *STS*, Society of Thoracic Surgeons.

### Intraoperative Data

Intraoperative findings are summarized in Table 2. More than one half of the operations were elective, 6 were urgent and 2 emergent. All cases were performed through conventional full sternotomy. Moreover, 42% of surgeries were done in an urgent/emergent/salvage setting, for which conventional sternotomy was preferred. There was no reported intraprocedural mortality.

**Table 2:** Descriptive analysis of intraoperative variables

Variable	Value
Admission status, n (%)	
Elective	11 (57.9)
Urgent	6 (31.6)
Emergent	2 (10.5)
Conventional full sternotomy, n (%)	19 (100)
MV procedure, n (%)	
MV repair	1 (5.3)
MV replacement, biological valve	18 (94.7)
MV replacement, mechanical valve	0 (0)
Concomitant procedures, n (%)	
TAVR explant	10 (52.6)
AV replacement, n (% of TAVR explants)	
Biological valve	10 (100)
Mechanical valve	0 (0)
CABG	2 (10.5)
TV repair	4 (21.1)
ECMO implantation	2 (10.5)
LAAL	8 (42.1)
ASD/PFO closure	5 (26.3)
Septal/left ventricular myectomy	3 (15.8)
Number of concomitant procedures, n (%)	
0	2 (10.5)
1	4 (21.1)
2	5 (26.3)
3 or more	8 (42.1)
Intraoperative blood products use, n (%)	19 (100)

*MV*, Mitral valve; *TAVR*, transcatheter aortic valve replacement; *AV*, aortic valve; *CABG*, coronary artery bypass graft; *TV*, tricuspid valve; *ECMO*, extracorporeal membrane oxygenation; *LAAL*, left atrial appendage ligation; *ASD*, atrial septal defect; *PFO*, patent foramen ovale.

Replacement with a bioprosthetic valve represented the preferred surgical strategy adopted, with only 1 patient with functional MR who underwent repair with an annuloplasty device. TAVR explant was performed in 10 (52.6%) cases, and the AV was replaced with a tissue prosthesis. Concomitant procedures included coronary artery bypass graft (n = 2), tricuspid valve repair (n = 4), exclusion of the left atrial appendage (n = 8), closure of atrial septal defect or patent foramen ovale (n = 5), septal myotomy/myectomy (n = 3). Two patients required intraoperative extracorporeal membrane oxygenation (ECMO) for postcardiotomy syndrome.

### Postoperative Data

Postoperative data are shown in Table 3. Five patients died within 30 days (26.3%), all during the hospital stay, and 1 of them had a stroke. One patient required ECMO for cardiogenic shock. Median CPB and XCT were 214 (155, 253) minutes, and 144 (106, 189) minutes, respectively. Total ICU and in-hospital lengths of stay were 120.4 (55.4, 440.4) hours and 21.3 ± 11.2 days, respectively.

**Table 3:** Descriptive analysis of postoperative variables

Variable	Value
Primary end points,∗ n (%)	5 (26.3)
Mortality, n (%)	
Intraoperative mortality	0 (0)
In-hospital mortality	5 (26.3)
Cerebrovascular accidents, n (%)	1 (5.3)
Secondary end points†	
CPB, min, mean ± SD	214 (155, 253)
XCT, min, mean ± SD	144 (106, 189)
OR use time, min, median (IQR)	466 (423, 545)
ICU total length of stay, h, median (IQR)	120.4 (55.4, 440.4)
In-hospital length of stay, d, mean ± SD	21.3 ± 11.2
Postoperative renal failure requiring new HD, median (IQR)	4 (21.1)
Postoperative creatinine, mmol/L, median (IQR)	1.3 (1.09, 2.9)
Perioperative mortality in patients requiring new HD, n (%)	4 (100)
Prolonged ventilation (>24 h), n (%)	8 (42.1)
Ventilation, h, median (IQR)	17.8 (9.9, 196.6)
Reintervention due to cardiac causes, n (%)	1 (3.7)
Rehospitalization, n (%)	3 (15.8)

*CPB*, Cardiopulmonary bypass time; *SD*, standard deviation; *XCT*, crossclamp time; *OR*, operating room; *IQR*, interquartile range; *ICU*, intensive care unit; *HD*, hemodialysis.

∗ Primary end points: all-cause mortality and stroke at 30 d.

† Secondary end points: CPB, XCT, and OR use time, ICU and in-hospital lengths of stay, postoperative renal failure requiring dialysis, prolonged ventilation (>24 h), reintervention attributable to cardiac causes, and rehospitalization.

Among the 4 patients who required hemodialysis for postoperative oliguric renal failure, 3 died in-hospital. Total median mechanical ventilation time was 17.8 (9.9, 196.6) hours, with 8 patients requiring prolonged ventilation (>24 hours). Among the 14 patients who were discharged, 3 were readmitted for heart failure exacerbation and were successfully managed medically.

Three of the 5 patients who died had their TAVR explanted; their causes of death were the following: 1 patient went into right ventricular failure and cardiogenic shock after the procedure, ultimately dying on postoperative day 4; another patient died on postoperative day 19, after a prolonged ICU course, complicated by renal failure requiring hemodialysis, complete heart block and toxic metabolic encephalopathy; the third demise happened on postoperative day 29, with a postoperative course complicated by a tension pneumothorax, worsening right ventricular dysfunction and cardiogenic shock requiring institution of peripheral VA ECMO (postoperative day 23), and septic shock. The remaining 2 patients who died before discharge did not undergo a TAVR explant; their causes of death were as follows: 1 of the 2 patients died on postoperative day 29 because of cardiac cardiogenic shock, respiratory failure with prolonged intubation and renal failure requiring hemodialysis; the last death was caused by multiorgan failure on postoperative day 27, after combined septic and cardiogenic shock, prolonged ventilation followed by tracheostomy for respiratory failure and hemodialysis for renal failure. Especially in cases that required a TAVR explant, both the length and the difficulty of the procedure, in addition to the morbid features of this patients’ cohort, contributed to their poor outcomes.

## Discussion

Surgical management of MV disease in patients with previous TAVR is challenging. We report a single center retrospective analysis on the outcomes of 19 patients with TAVR who underwent MV surgery at Columbia University Medical Center from July 1, 2014, to July 1, 2024.

The key takeaways from this study are the following: (1) MV surgery in the presence of a THV carries a significant 30-day morbidity and mortality. (2) Degenerative and infectious disease are the most common indications for MV surgery post-TAVR. (3) A significant proportion of patients requires concomitant TAVR explantation, a procedure associated with a high rate of mortality and complications.

MV surgery post-TAVR is an uncommon scenario, because we identified only 19 patients out of a total of 2339 MV operations (0.85%) in 10 years. The most likely explanation for the low prevalence is the development of transcatheter techniques for the treatment of both degenerative and functional MR. Specifically, if the patient already underwent a TAVR because they were deemed a suboptimal candidate for surgery, percutaneous transcatheter therapies for MV therapy continue to represent their first line of treatment. Edge-to-edge repairs and TMVRs are available options for these patients, unless there is any anatomical contraindication. Surprisingly, the median time from the TAVR to the MV surgery was only approximately 1 year. Excluding 7 patients with IE, the 12 patients included in our analysis did not meet any criteria for MV intervention at the time of TAVR.

### Risk Stratification and Patient Selection

Although TAVRs are performed in patients of all risk profiles, a significant percentage of them are offered to frail, elderly patients that are at high risk for surgery. In our series, 2 patients had a history of chest radiation, and 9 had previous cardiac surgical interventions, predicting an increased risk of chest re-entry. Mean preoperative Society of Thoracic Surgeons predicted risk of mortality was 12.0 ± 9.3%, identifying a high-risk cohort. Our observed-over-expected 30-days mortality ratio was greater than 1.0, similarly to the existing literature on cardiac operations after TAVR.^12^^,^^13^ The involvement of the aortic prosthesis in the disease process, or the need for TAVR explant in addition to the mitral repair or replacement, significantly increases the surgical risk.^17^ In patients requiring multiple valve procedures, especially if necessitating THV explantation, CPB and XCT were notably longer, as well as time of mechanical ventilation, ICU length of stay, and hospital length of stay.

The risk profile of this category of patients is different than the one with no previous cardiac procedures. Although TAVRs are nowadays performed in patients of all risk categories, a significant percentage of them are offered to old and sick patients that are high risk for surgery. This being said, if these patients ever need MV surgery after their TAVR, their procedural risk would be at least the same or likely greater than before. Therefore, the surgeons would have to perform more arduous interventions in a sicker patient population. Moreover, the involvement of the aortic prosthesis in the disease process, or the need for TAVR explant in concomitance to the mitral repair of replacement, would shift the surgery in a totally different risk category.

Isolated TAVR explant is known to be a technically demanding procedure, frequently requiring extensive cardiac reconstruction. Data from the TAVR-EXPLANT registry were not promising.^18^ In this cohort of intermediate-risk patients, the 30-day mortality and strokes rates were 13.1% and 8.6%, respectively, and almost one third died at 1 year. After the promising results of recent trials,^19^, ^20^, ^21^, ^22^ more patients with a lower risk profile undergo TAVR for isolated AV disease. Arguably, as the age of the general population continues to increase, the incidence of de novo MV disease in these patients will increase as well, and concomitant MV surgery and THV explantation will be more common. In the TAVR-EXPLANT registry, one third of the patients had concomitant MV intervention, mirroring the cohort of patients described in our study, and mitral surgery was shown to be a predictor of risk-adjusted early and late mortality; in addition, duration of CPB, XCT, ventilation time, and pulmonary hypertension were associated with decreased survival ^18^We suggest that age as well as the presence of any type of MV disease at the time of first-time AV intervention should be carefully assessed by the surgical and TAVR teams, with consideration of the difficulty as well as the morbidity and mortality associated with a surgical operation post-TAVR. Nevertheless, because MV pathology is not deemed significant at the time of the aortic intervention, it is hard to address it instead of performing an isolated AV replacement at the index procedure.

### Mitral Valve Interventions

#### Transcatheter options

**Figure 2:**
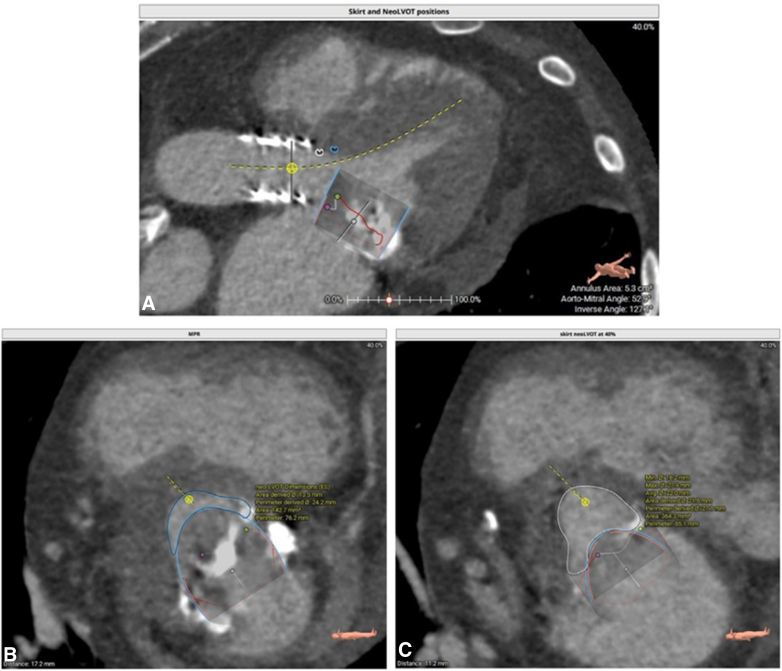
LVOT obstruction after transcatheter mitral valve replacement is a possible complication of transcatheter mitral valve replacement. A, CT reconstruction of LVOT in a patient with a THV with virtual implantation of SAPIEN valve in the mitral position. B, Assessment of neo-LVOT. C, Skirt-to-neoLVOT in 40% phase. *CT*, Computed tomography; *LVOT*, left ventricular outflow tract; *THV*, transcatheter heart valve.

In high-risk patients with MV disease who underwent TAVR, transcatheter options should be considered as first-line therapy. TEER with commercially approved clip devices is an established therapy for both primary and secondary MR. Nevertheless, calcified or severely thickened leaflets, multiple complex regurgitant jets, mitral annular calcification, elevated baseline gradients and small valve areas, commissural flail segments, short leaflets, and wide regurgitant gaps are relative contraindications to TEER intervention. The use of TMVR devices is limited by commercial availability and anatomical constraints such as risk of LVOTO and annular size, representing 2 of the most frequent concerns and reasons for exclusion from enrollment in trial devices. In case of MV IE, the role for transcatheter therapy is clearly limited. In our series, MS, the risk of LVOTO (Figure 2),^23^ and endocarditis, excluded these patients from consideration of any commercial or investigational transcatheter MV replacement devices.

#### Surgical options

MV surgery in patients with previous TAVR reveals potentially significant challenges, including exposure of the MV, sewing the new bioprosthesis or annuloplasty device, and the potential need for THV explant and surgical AV replacement. With a TAVR in place, visualization and exposure of the anterior mitral annulus and MV is often difficult. Furthermore, the retraction of the left atrial roof for valve exposure may deform and damage the THV stent frame leading to AR. The deeper a TAVR valve is implanted below the annular plane, the closer it is to the anterior mitral leaflet, interfering with the leaflet motion and difficulty in exposure. If the metal frame is permanently damaged and indented by the atrial retractor, the THV might remain deformed, resulting in worsening AR.

### MV Surgery With Normal Functioning THV

A common dilemma is what strategy to adopt at the time of MV surgery in the presence of a well-functioning THV. When the THV implant is relatively recent and does not show any sign of structural valve degeneration, the benefit of THV replacement with a new valve at the time of mitral surgery is unclear. The decision is determined on the basis of several factors such as the age of the THV implant along with clinical and echocardiographic criteria.^24^ Different strategies may be used for such operations, from conventional full sternotomy, to minimally invasive^25^ and robotic surgeries.^26^ The type of approach may vary, depending on the indications for the surgery, as well as patient's and surgical team's preferences.

The variability in durability of the THVs depends on intrinsic valve related factors (eg transvalvular gradients, prosthesis-patient mismatch, rate of underexpansion, depth of implantation, etc), as well as on patient characteristics, genetics, biological factors and comorbidities (eg, renal insufficiency). An older individual with significant comorbidities who undergoes MV surgery in the presence of a functioning THV, should probably be spared from an additional procedure that would increase the risk of the operation. If the patient is a candidate for redo-TAVR, surgeons may defer the treatment to the time the THV shows signs of structural valve failure. The decision to address and replace a recently implanted THV is determined by surgeon assessment, as guidelines do not yet exist. A common reason to explant a functioning THV is to improve exposure to the MV, especially the anterior annulus and the trigones. Second, despite no long-term data on durability of TAVR valves, it is reasonable to replace the AV if implanted more than 5 years previously, especially in younger patients, or in those with no option for THV-in-THV on the basis of preoperative computed tomography assessment. Among our patients who had their TAVR explanted, 10 showed signs of dysfunction and 5 showed signs of IE.

### MV Surgery With Degenerated THV

If the THV shows signs of structural valve degeneration, the decision to replace is easier. TAVR-explant techniques are being developed and streamlined. Strategies like the “double Kocher” or the “roll” techniques for BEVs and the “tourniquet” and “handlebar and mustache” for SEVs are being implemented by specialized aortic surgeons in high-volume centers.^27^ Of the 10 explanted TVH in our study, 2 were planned as they showed signs of greater-than-moderate AS/AR, whereas the remaining 8 were performed to improve the surgical exposure to the MV. Among the 5 patients that deceased, 3 had their THV explanted at the time of surgery (Video 1).

### Limitations

The main limitation of this analysis is the small number of subjects included. This is a single-center retrospective study whose results could not be generalized. Nineteen patients over a 10-year period shows that this is an uncommon scenario. Also, long-term follow-up is not complete, and we are not aware of the clinical conditions of all patients after discharge.

## Conclusions

With widespread use of TAVR for the treatment of AV stenosis, patients may develop significant MV disease that requires therapy. If no transcatheter options are suitable, or in case of IE, MV surgery may need to be undertaken. Performing isolated MV surgery without interfering with the THV is not feasible in all cases. TAVR explant increases the risk and complexity of the mitral intervention. Our limited analysis on patients with TAVR undergoing MV surgery shows that this procedure is associated with high mortality and morbidity, with an observed-over-expected mortality ratio greater than 1.0. The development of novel transcatheter devices will likely provide less-invasive alternatives to surgery, providing nonsurgical options in patients with degenerative or functional mitral pathology.

## Conflict of Interest Statement

The authors reported no conflicts of interest.

The *Journal* policy requires editors and reviewers to disclose conflicts of interest and to decline handling or reviewing manuscripts for which they may have a conflict of interest. The editors and reviewers of this article have no conflicts of interest.

## Supplementary Data

Video 1 Explanation of self-expandable transcatheter heart valve prior to MV replacement. Video available at: https://www.jtcvs.org/article/S2950-6050(25)00022-1/fulltext.
