# Supplementary figures and images for: Mitral valve surgery post-transcatheter aortic valve replacement: A 10-year, single-center, retrospective analysis
Source: JTCVS Struct Endovasc. 2025 Jul 16;7:100063. doi: 10.1016/j.xjse.2025.100063 (PMC13244723; doi:10.1016/j.xjse.2025.100063)

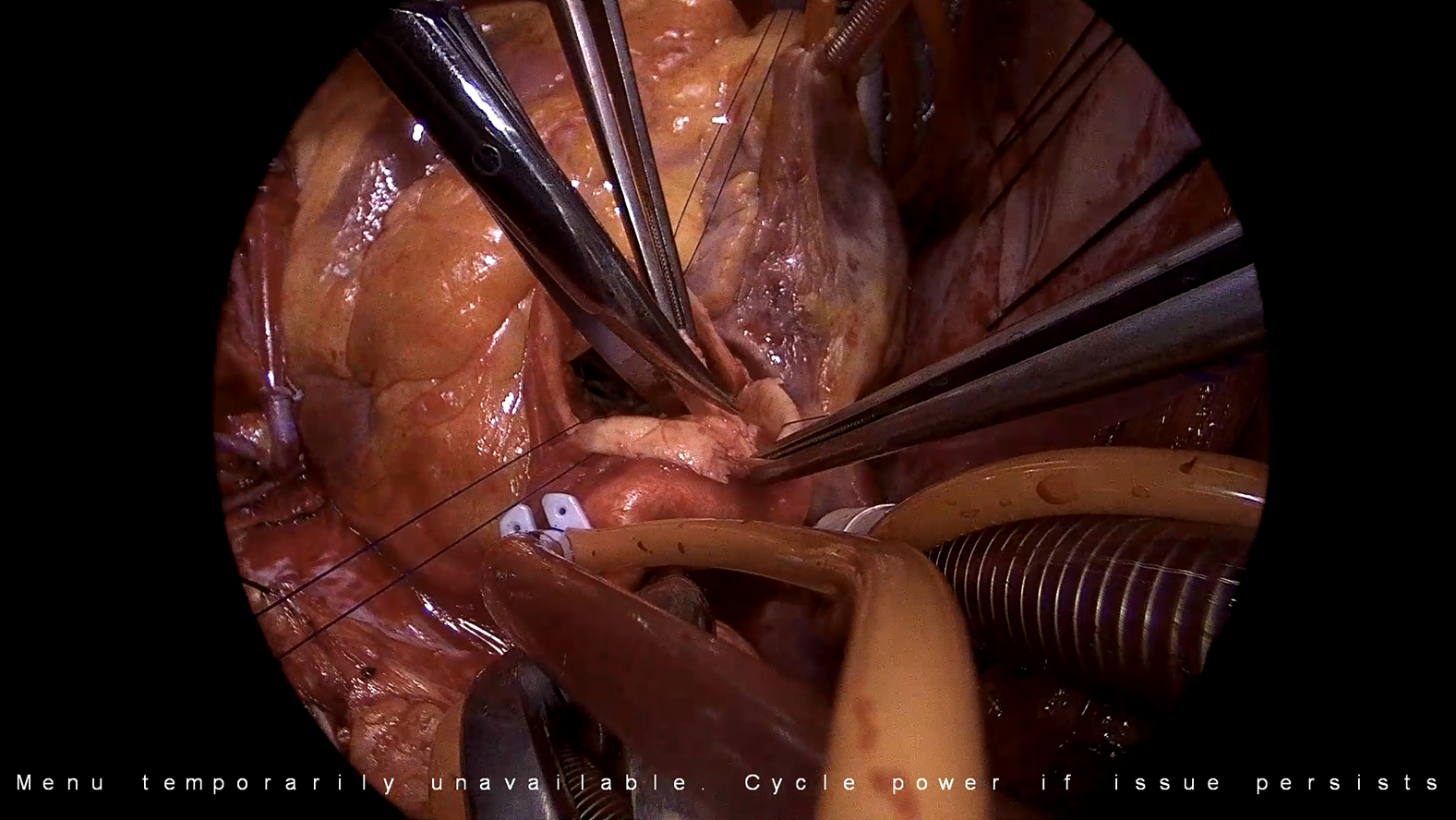

Supplement: Video 1 — Explanation of self-expandable transcatheter heart valve prior to MV replacement. Video available at: https://www.jtcvs.org/article/S2950-6050(25)00022-1/fulltext. [file fx2.jpg]
